# Effect of site-directed mutagenesis at the GGEEF domain of the biofilm forming GGEEF protein from *Vibrio cholerae*

**DOI:** 10.1186/s13568-015-0168-6

**Published:** 2016-01-04

**Authors:** Om Prakash Chouhan, Divya Bandekar, Mousumi Hazra, Ashish Baghudana, Saugata Hazra, Sumit Biswas

**Affiliations:** VISTA Lab, BITS, Pilani, KK Birla Goa Campus, Zuarinagar, Goa 403726 India; Department of Microbiology, University of Kalyani, Kalyani, West Bengal 741245 India; Department of Biotechnology, Indian Institute of Technology Roorkee, Roorkee, Uttarakhand 247667 India

**Keywords:** Diguanylate cyclase, Phosphodiesterase, Biofilm, Motility, Site directed mutagenesis

## Abstract

*Vibrio cholerae*, the cause of seven noted pandemics, leads a dual lifecycle—one in the human host in its virulent form, and the other as a sessile, non-virulent bacterium in aquatic bodies in surface biofilms. Surface biofilms have been attributed to be associated with a ubiquitous protein domain present in all branches of bacteria, known as the GGD(/E)EF domain. While the diguanlyate cyclase activities of these proteins are universally established, the role of these proteins as diguanlyate-specific phosphodiesterases in conjunction with a EAL domain has also been reported. The VC0395_0300 protein from *V. cholerae* which shows biofilm forming abilities also acts as a phosphodiesterase. Interestingly, this GGD(/E)EF protein contains a EAL site in the reverse orientation. We attempted to mutate the GGEEF signature along the sequence by site-directed mutagenesis. The resultant mutants (Sebox5–7) did not show much difference in phosphodiesterase activity in comparison with the wild type protein (Sebox3), indicating the independence of the phosphodiesterase activity of the protein from the GGD(/E)EF domain. However, the ability of the mutants to form surface biofilm was significantly lesser in the case of mutations in the three central positions of the signature domain.

## Introduction

The Gram negative flagellate *V. cholerae* survives as a non-motile, non-virulent, biofilm in aquatic bodies in between cholera epidemics. Biofilms are resistant to external stress like antibiotics, chlorine, predators and other factors (Ryjenkov et al. 2005; Hammer and Bassler 2009). Biofilm-forming bacteria have been shown to secrete an external exopolysaccharide matrix (Karatan and Watnick 2009). Proteins with a conserved GGD(/E)EF domain have been implicated in the regulation of the polysaccharide production in *Salmonella typhimurium*, *Pseudomonas aeruginosa* and almost all eubacterial species. A number of these GGD(/E)EF putative domains are also present in the *V. cholerae* genome as well, distributed across both chromosomes. GGD(/E)EF domain proteins have mostly been shown to synthesize (as diguanylate synthases) the bacterial secondary messenger cyclic-di-GMP (c-di-GMP), whereas enzymes having an EAL or HD-GYP domain (phosphodiesterases) generally degrade the messenger (D’Argenio and Miller 2004; Tischler and Camilli 2004; Ryan et al. 2006; Yan and Chen 2010; Hengge 2013). It has been accepted that an increase in the levels of c-di-GMP is usually associated with the formation of the exopolysaccharide matrix and the surface biofilm in *V. cholerae.* On the other hand, a decrease in c-di-GMP concentrations due to phosphodiesterase (PDE) activity leads to the bacterium showing functional flagella and consequent loss of biofilm (Lim et al. 2007; Waters et al. 2008).

Another common observation has been the coupling of these domains, viz., the GGD(/E)EF, HD-GYP and EAL domains with sensory domains like the PAS, GAF and DICT, suggesting a role for these proteins in sensing environmental signals (D’Argenio and Miller 2004). However, the exact mechanisms of this synergy has not been deciphered yet as different ligands have been known to mediate the signaling process for different proteins. Additionally, it is an observed fact that a single bacterium may possess multiple putative proteins which code for GGD(/E)EF or EAL or both. While *Escherichia coli* K12 codes for 12 proteins with GGD(/E)EF, 10 with EAL and 7 with both, *P. aeruginosa* encodes 17 GGD(/E)EF, 5 EAL and 16 with both. Likewise, *V. cholerae* codes for 31 GGD(/E)EF, 22 EAL and 10 with both (Lim et al. 2007; Tamayo et al. 2007; Waters et al. 2008; Yan and Chen 2010). However, mutation in a single protein can result in disruption of morphology for the bacterium. How or why this multiple coding system has been sustained and why morphological changes happen due to mutation in a single protein have also been subjects of intense debate.

Essentially, a diguanylate cyclase has been shown to have two active sites (Castiglione et al. 2011). The catalytically active A-site with a conserved GGD(/E)EF loop binds one monomer of GTP, which is the substrate. When two such substrate-loaded monomers come together in an antiparallel orientation, the synthesis of c-di-GMP takes place. The other site, the I-site, is known to bind the c-di-GMP and is often associated with the allosteric inhibition of diguanylate cyclase activity (Gao and Stock 2009; Yang et al. 2011; Marmont et al. 2012). Therefore, a majority of the proteins with the GGD(/E)EF signature sequence have been found to possess diguanylate cyclase activity. On the other hand, there have been reports of proteins having unorthodox GGDEF signature sequences and therefore, lack of diguanylate cyclase activity. However, these proteins have still retained the ability to bind GTP at the active site (Christen et al. 2005).

The putative GGD(/E)EF protein VC0395_0300 from the chromosome I of *V. cholerae* classical strain O395 shows the presence of a GGD(/E)EF domain, as well as a PAS domain signature, as determined from domain prediction software. This protein was overexpressed as a recombinant system in *E. coli* and purified. Subsequent determination of activity revealed the lack of diguanylate cyclase activity for the protein, but demonstrated the presence of phosphodiesterase activity. However, the VC0395_0300 protein was shown to have a distinct role in biofilm formation. Site-directed mutagenesis at the central GEE sites of VC0395_0300 generated mutants, all of which lacked biofilm forming ability. The phosphodiesterase activity was preserved in all the mutants; additionally, none of them showed any diguanylate cyclase activity. Interestingly, the VC0395_0300 protein does not possess an ‘EAL’ or ‘HD-GYP’ domain in its sequence, but an EAL signature in reverse (LAE), which is an aberration because all known phosphodiesterases reported till now have been shown to be associated with an ‘EAL’ or ‘HD-GYP’ signature.

## Materials and methods

### Bacterial strains and plasmids

Genomic DNA from *V. cholerae* classical strain O395 was extracted and purified according to the method in (Bhuiyan et al. 2012). *E. coli* BL21 (DE3) and DH5α were procured from Novagen. The gene encoding VC0395_0300 (putative GGDEF protein) from *V. cholerae* was PCR amplified with *DreamTaq* DNA polymerase (Fermentas) and the following primers: Sebox 1A (*Bam*HI, *Xho*I) 5′AATACTGGATCCATGAAAAATTGGCTGTG TCAGGCAGTG 3′ and 5′AATACT CTCGAGTTATTCTGTGGATTGGCGATAGATACA 3′. The restriction enzyme names are within parentheses, and their sequence in the primers indicated as underlined. For site-directed mutagenesis, the following primers were utilized: Sebox 5 (new_mutant at position 2 generating the second G): 5′ GATGATGAACTC TTCAC*g*TCCCACACG 3′; Sebox 6 (new mutant at position 3 generating the first E): 5′ GATGATGAACTCTT*t*ACCTCCCACACG 3′; Sebox 7 (new_mutant at position 4 generating the second E): 5′ GATGATGAACT*t*TTCACCTCCCACACG 3′. The site of the mutation has been shown in italics in the above three primers. The amplified fragments were purified using QiAquick kit from Qiagen, and digested using restriction enzymes from Fermentas. This was cloned into the corresponding sites of the vector pGEX-6P-1 (GE Healthcare) in *E. coli* DH5α.

### Protein expression and purification

VC0395_0300 was expressed as a recombinant GST-fusion product in *E. coli* BL21 (DE3). A 2 litre bulk culture was induced by addition of 0.05 mM IPTG (final concentration) for 8 h at 16 °C and 180 rpm. The cells were then harvested by centrifugation and suspended in lysis buffer [50 mM Tris–HCl (pH 7.4) and 50 mM NaCl]. The suspension was lysed by sonication with low amplitude pulses of 30 s duration each, followed by centrifugation to separate the insoluble fractions. The supernatant was loaded into glutathione agarose resin (GE Healthcare Life Sciences), which had been pre-equilibrated in a compatible buffer, for 1 h at 4 °C. The target protein fractions were eluted using an elution buffer containing 10 mM reduced glutathione and the purity of the protein was checked on a 10 % Tris–glycine SDS PAGE under denaturing conditions. The purified fractions were pooled and dialysed against a Tris buffer of pH 8.0. Protein concentration was determined using Bradford reagent (Bradford 1976) at each step.

### Cleavage of the GST tag

The GST tag being a 26 kDa fusion protein made it essential for the tag to be removed for further characterization of the GGEEF protein. Hence the tagged protein isolated previously was reloaded into the GST Sepharose column according to the conditions listed in the manufacturer’s protocol (Harper and Spiecher 2011). The resin-bound protein was treated with 10 units/mg of PreScission Protease (GE Healthcare Life Sciences) at 4 °C overnight. Subsequent washing and elution of the column resulted in purified VC0395_0300 bereft of GST tag. Similar method was employed for the extraction, purification and cleavage of the three mutants as well.

### Biofilm formation and quantification assays

Biofilm formation assay was performed according to the method described previously (Boyd and O’Toole 2012). Briefly the method encompassed inoculation of fresh 5 ml LB broth in glass tubes (18 × 150 mm) containing antibiotic with 1 % overnight bacterial cultures and incubation at 37 °C with shaking at 120 rpm for 12 h. Thereafter, all the tubes were transfer to static culture conditions for incubation. The tubes were observed for the appearance of thin slimy film or pellicles at the air–liquid interface. Upon removal of all the liquid medium from the test tubes, the bacterial cells bound to the walls were stained with 0.2 % crystal violet for 5 min at room temperature. Unbound excess crystal violet was removed by rinsing the tube with distilled water. Tubes were air dried at room temperature and photographed. This was replicated for multiple sets for each day starting from 1 day to 7 days. For spectrophotometric quantification, the biofilm was dissolved in 4 ml of 75 % ethanol and absorbance noted using a spectrophotometer at 570 nm.

### Motility assays

Motility test was performed utilizing the color conversion of triphenyl tetrazolium chloride (TTC) as described in previous reports (Ball and Sellers 1966; An et al. 2010). In its oxidized form, TTC is a colorless compound that is absorbed into the bacterial cell which grows in its presence. Here, TTC is reduced to form a red colored pigment called formazan. To perform motility assay, single isolated colony was deep stabbed to a LB agar tube containing TTC using a sterile inoculating needle and incubated at 37 °C until growth was evident. Bacterial growth is indicated by the appearance of a red color formed due to the production of formazan.

### In vitro phosphodiesterase activity assays

Purified wild type Sebox3 and the mutated strains (Sebox5, Sebox6 and Sebox7) were assayed for in vitro phosphodiesterase activity. The protein concentration was kept uniform after performing Bradford assay. PDE activity was performed using chemically synthesized substrate bis(*p*-nitrophenyl) phosphate (obtained from Sigma Aldrich) using standard protocol (Liu et al. 2010) with some minor changes. 50 μl of reaction mixture was prepared consisting of 50 mM Tris–HCl (pH 7.4), 10 mM MnCl_2_, 5 mM bis-pNPP to which was added 50 μl of the protein sample. The reaction mixture was mixed well and incubated for 8 h at 37 °C. The amount of released *p*-nitrophenol was detected and quantified by a Shimadzu UV–Vis spectrophotometer at an OD of 410 nm. Controls were set up without the addition of the protein and enzyme activity was compared in triplicate individual assays to ascertain the reproducibility of the reaction. Benziman had reported that Ca^2+^ strongly inhibited the phosphodiesterase activity in *Gluconacetobacter xylinus* (Ross et al. 1986, 1990). Therefore, we added 10 mM CaCl_2_ to the reaction mixture to check for Ca^2+^ induced inhibition in the wild type as well as mutant samples.

### Fluorescence spectroscopy

To estimate the position of the tryptophans (two in number) present in the wild type and mutants, fluorescence studies were carried out in a JASCO FP8200 spectrofluorimeter. Both the excitation and emission band passes were kept at 5 nm. For the denaturation study, a series of fresh solutions of guanidinium hydrochloride (GdnHCl) having concentrations in the range of 0.5–5 M in 50 mm Tris HCl buffer (pH 8.0) were prepared and the proteins were added to a final concentration of 7 μM. Proteins were incubated separately in GdnHCl containing buffer overnight at 25 °C. Equal volumes of buffer were added to the same volume of GdnHCl solutions and these mixtures were used as blank. The excitation wavelength was kept at 295 nm and the emission spectra were noted.

### Comparison of homology models

A BLAST query was performed with the sequence of VC0395_0300 against all PDB structures to find homologues. The protein from *Marinobacteraquaeolei* diguanylate cyclase complexed with c-di-GMP (pdb id. 3ign) was the best match in terms of query coverage and E-value. There was approximately 40 % sequence similarity between the sequences of VC0395_0300 and 3ign. A homology model of VC0395_0300 was constructed using the Modeller Suite (Version 9.1). The initial model was further improved by energy minimization using GROMACS 4.5 (Berendsen et al. 1995; Abraham and Gready 2011) software package. After the optimization procedure, minimized model was validated using PDBsum where the evaluation was based on standard bond length, bond angle, Ramachandran plot, etc. (Laskowski 2001). A similar method was adopted for all the three mutants and the modeled structures of Sebox 3, 5, 6 and 7 were compared for the surface architecture of the GGEEF region.

## Results

**Fig. 1:**
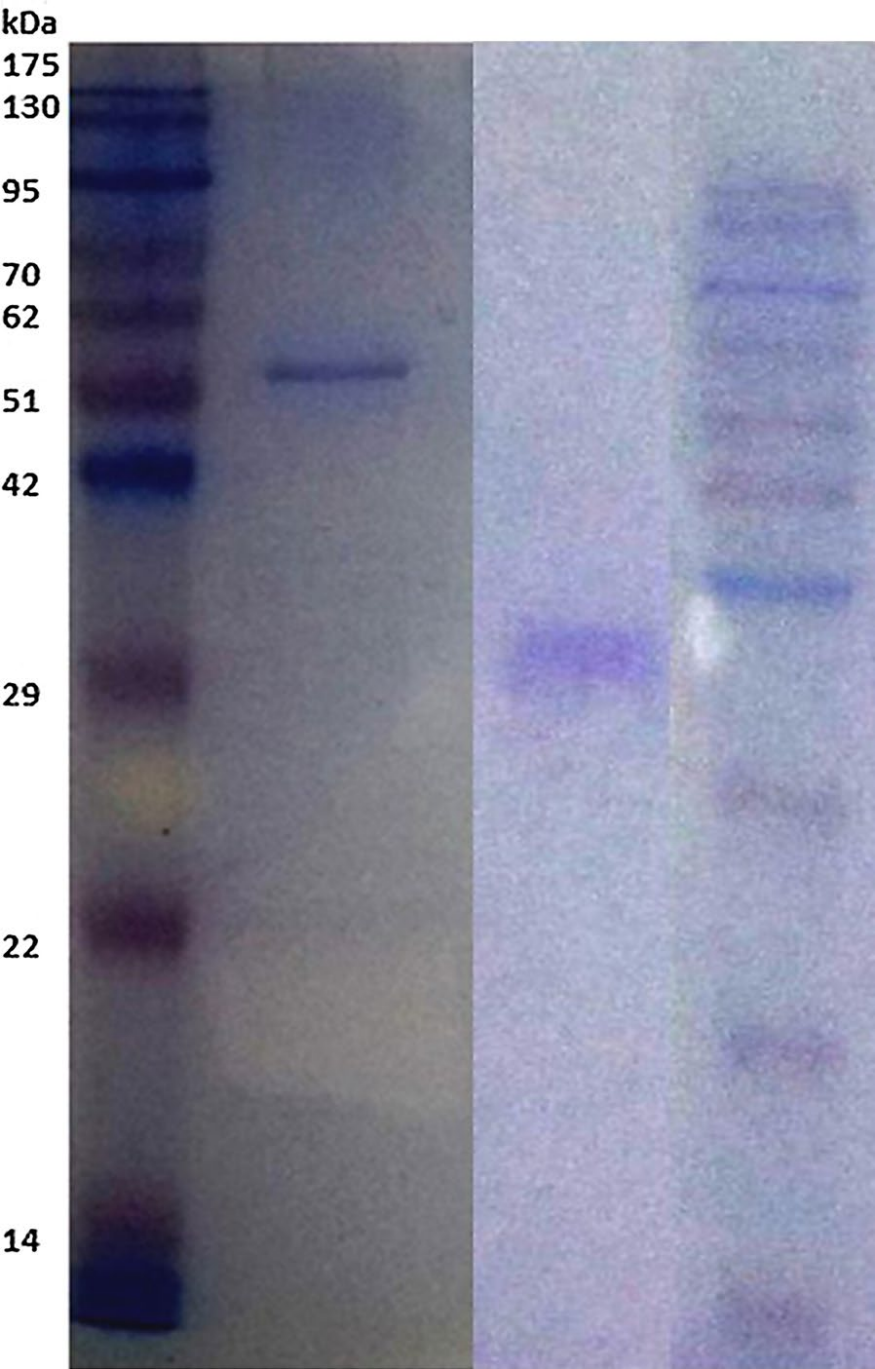
Purification profile of tagged Sebox5 (*left*) and tagless Sebox5 (*right*) against pre-stained molecular weight ladder

Low cell viability has been reported for GGD(/E)EF proteins previously expressed from various sources (Ryjenkov et al. 2005). The same was evident for our clones as well, and transformation efficiency was abysmally low. During expression and extraction, care had to be taken for obtaining active protein in the soluble fractions. Initially, recombinant wild type Sebox3 was obtained in small quantity in the supernatant fractions and had to be standardized against buffer conditions to improve purity. The need for larger culture volumes was necessitated by the low yield of the protein after cleavage of tag. Similarly, for the mutants as well, yield needed to be standardized to achieve workable quantities (Fig. 1). Again, the tagless proteins were found to be unstable in solution.

### Mutation in the ‘GEE’ positions affects biofilm formation in *V. cholerae*

Site directed mutagenesis at the positions G, E and E of the GGEEF sequence in VC0395_0300 resulted in the generation of mutants with altered amino acids. While Sebox5 mutants had an arginine in place of the glycine, Sebox6 had the glutamate replaced by lysine, and Sebox7 also had a lysine in place of the glutamate. The generated mutants were checked by sequencing and also by blotting against anti-GST antibodies (GE Healthcare Life Sciences).

**Fig. 2:**
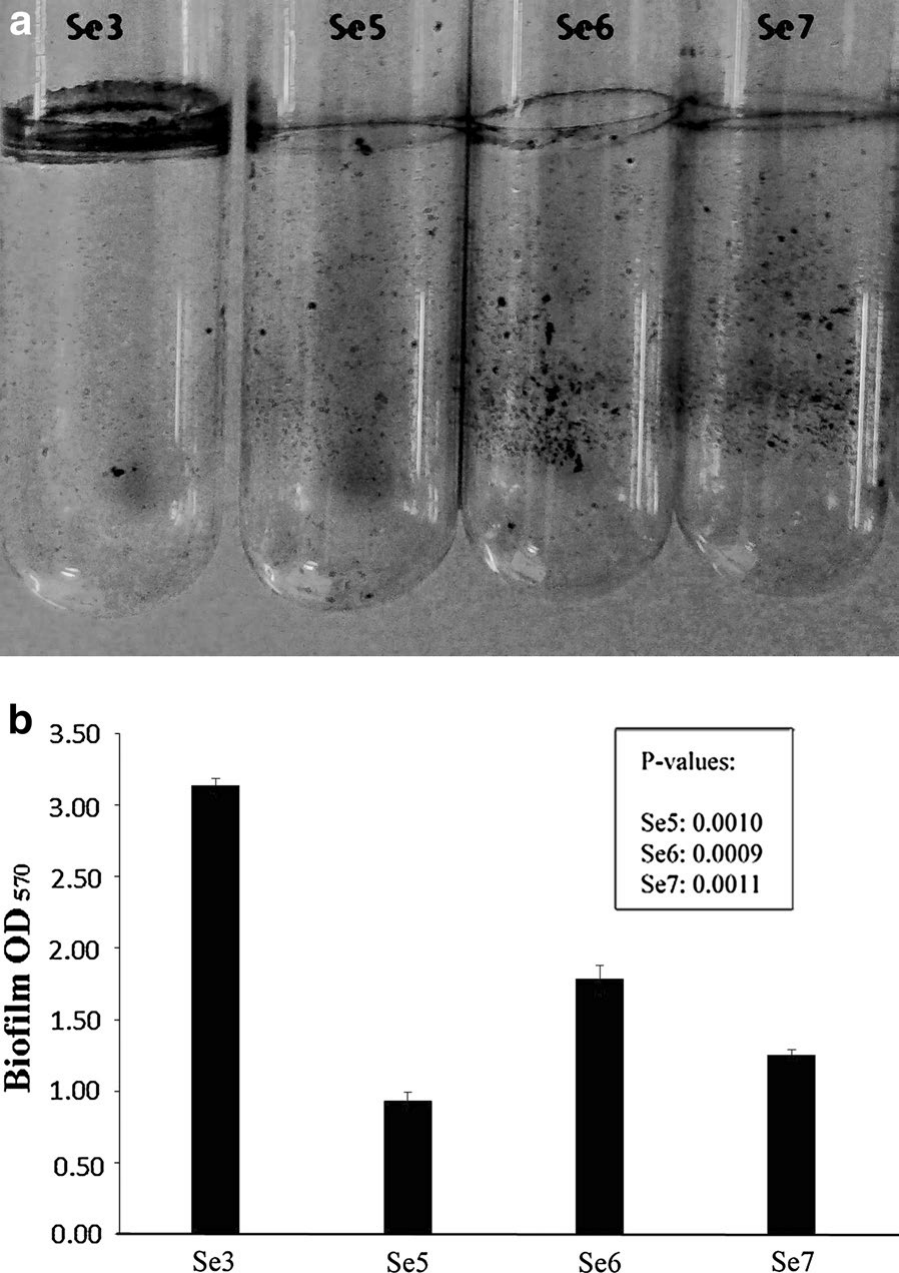
Biofilm formation in wild type Sebox3 and mutants Sebox5, 6 and 7. **a** Crystal violet stained biofilm in Sebox3 strains, lack of biofilm formation in Sebox5, 6 and 7. **b** Spectrophotometric estimation of biofilm formation by dissolving the biofilm formed in **a** and measurement of absorbance at a wavelength of 570 nm

Growth of the mutants in LB medium and transfer to static conditions for observation of biofilm formation was undertaken for separate sets at 2, 4 and 7 days intervals. After both 4 and 7 days, it was observed that the biofilm formation was significantly lower for the mutant Sebox5, 6 and 7 cultures when compared to the Sebox3 wild type. While the formation of biofilms decreased almost by threefolds in Sebox5 and 7, a twofold decrease was evident in Sebox6 (Fig. 2a, b).

**Fig. 3:**
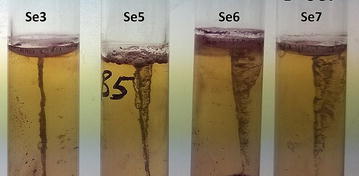
Assay for motility. Triphenyl tetrazolium chloride (TTC) forms a red colored compound, formazan, when absorbed into a bacterial cell. While Sebox3 shows almost no motility, Sebox5, 6 and 7 show migration from the initial stab line

The complementary motility assay was also performed to visualize the loss of biofilm formation abilities in the mutants. The TTC assay essentially marks out the migration of the bacterium from the zone of inoculation by the needle. After inoculation in the stab culture, the test tubes were allowed to incubate at 37 °C for 2 days without shaking. For the wild type strain Sebox3, there was no migration from the initial stab line, and no regions of color were visible. However, for the mutant strains, there was visible spread of the red formazan zone away from the site of inoculation of the initial stab (Fig. 3). All the three strains showed more-or-less comparable zone of motility, confirming our initial observations about loss of biofilm formation ability in the three mutants.

### The VC0395_0300 protein and the mutants do not have diguanylate cyclase activity but display in vitro phosphodiesterase activity

Since almost all GGD(/E)EF proteins display cyclic diguanylate activity, the proteins were initially assayed for the production of c-di-GMP, using the method employed previously (Ryjenkov et al. 2005). Briefly, a reaction mixture containing 5 μM protein, 10 mM MgCl_2_, and 50 mM NaCl was treated with 5 μM of GTP (Sigma Aldrich). The reaction mixture was prewarmed and the reaction was carried out for 0, 5, 15, 30, and 60 min. Aliquots were withdrawn at the given time intervals, heated in a boiling water bath for 3 min, 0.5 mM EDTA was added and centrifuged at 10,000*g* for 10 min.

The samples were injected into a X Terra RP18 column (250 mm × 4.6) from Waters and separated in an Agilent 1220 Infinity LC system for a total run time of 30 min at a flow rate of 0.6 ml/min. 20 mM triethyl amine in 9 % methanol and water was used as the solvent system for the process. NADP was added to the sample prior to injection as a standard. However, even after repeated runs with wild type and mutant proteins (at least four times for each), no peak corresponding to c-di-GMP was observed, indicating the absence of diguanylate cyclase activity in VC0395_0300 as well as its mutants.

**Fig. 4:**
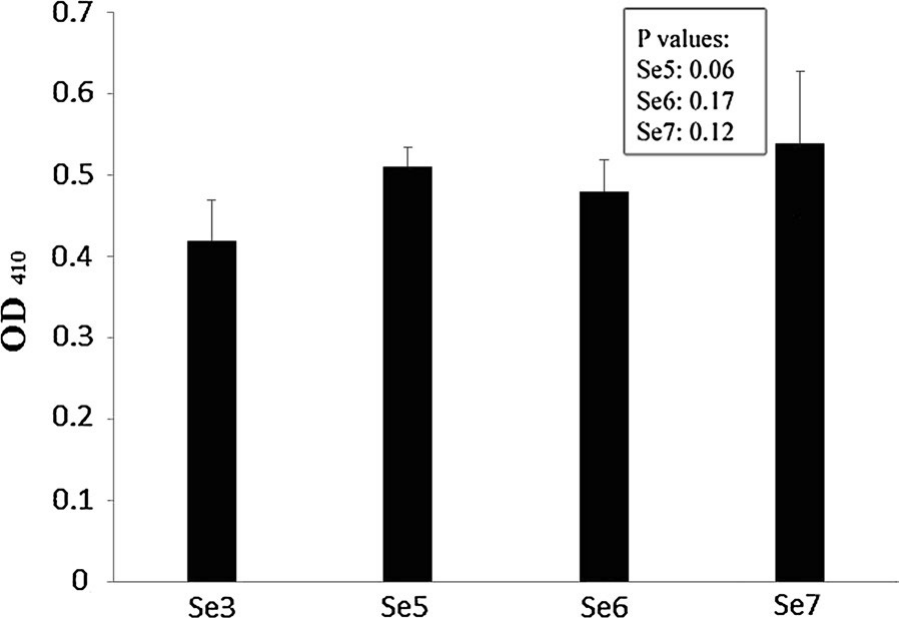
In vitro phosphodiesterase activity. Release of *p*-nitrophenol was measured at a wavelength of 410 nm after action of the enzyme on the substrate, bis(*p*-nitrophenyl) phosphate

The wild type and the mutants were both assayed for in vitro phosphodiesterase activity with bis(*p*-nitrophenyl) phosphate as the substrate. To our surprise, all the three mutants as well as the wild type demonstrated considerable activity with OD_410_ values ranging from an average of 0.41 (Sebox3) to 0.54 (Sebox7) (Fig. 4). Triplicate reactions confirmed the same for all four sets. Again, inhibition with CaCl_2_ reduced the OD_410_ value almost fourfolds indicating the inhibition of PDE activity by Ca^2+^, as had been the hallmark of other proteins having PDE activity. However, it is indeed notable that the VC0395_0300 protein does not have any EAL or HD-GYP domain in the correct orientation, ones that are usually associated with PDE activity. Since the mutants do not show any significant change in the PDE activity, it can also be safely concluded that the PDE activity is not associated with the GGEEF signature sequence.

### Estimation of changes in structure of mutants through fluorescence

**Fig. 5:**
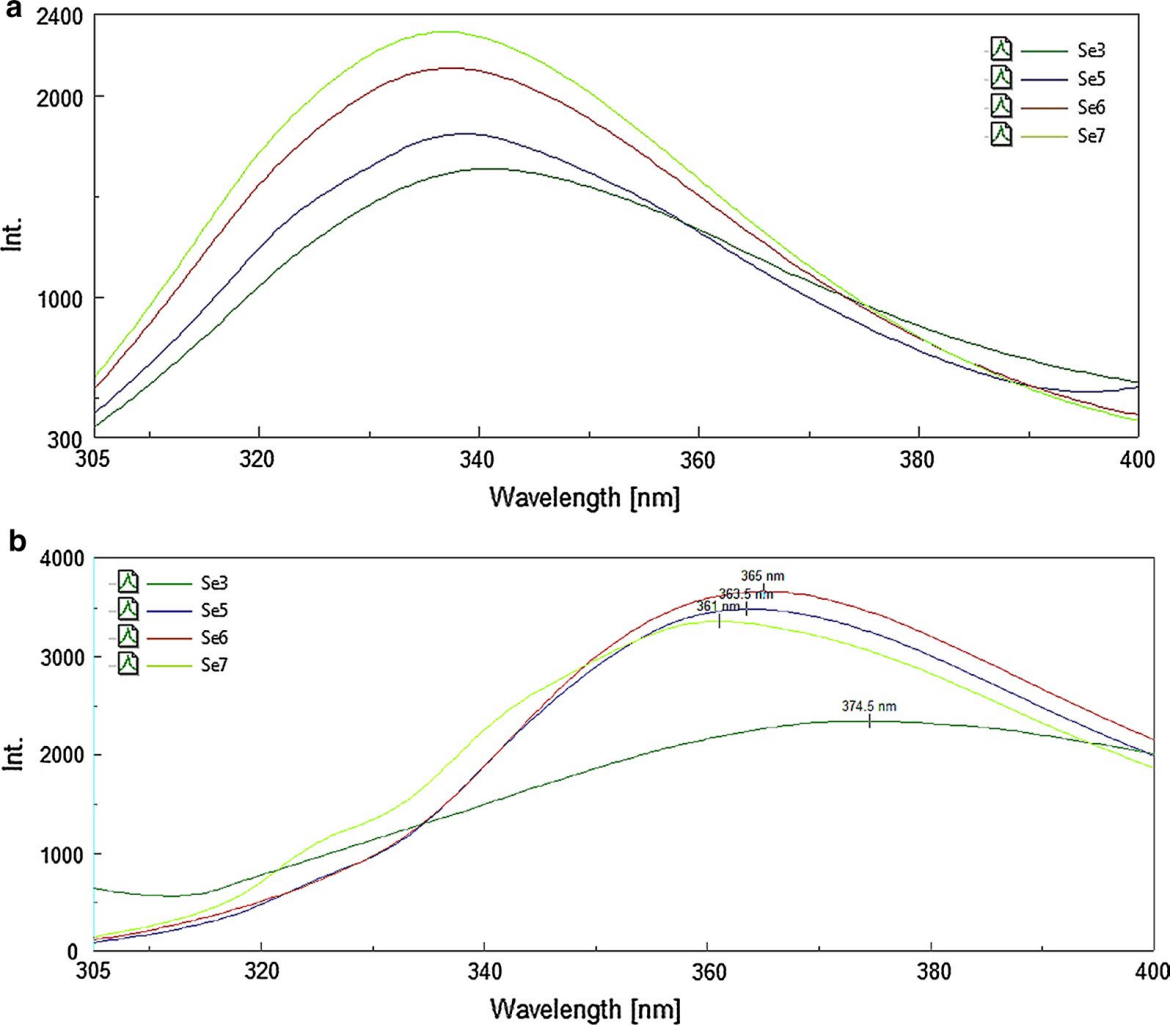
Shift in λ_max_ in the wild type and mutant proteins before **a** and after **b** complete unfolding with guanidinium hydrochloride

Intrinsic tryptophan fluorescence (λ_ex_ at 295 nm) was observed to infer any changes in the proteins’ architecture due to the effect of the mutations. The fluorescence spectra after denaturation with guanidinium hydrochloride showed a shift in the emission maxima (λ_max_) for both the wild type and mutant proteins (Fig. 5). While this shift was more pronounced in the case of the wild type protein, it was considerably less for the mutants. One of the tryptophans is within the first four amino acids from the N-terminal, and possibly would not be folded into the interior. The other tryptophan is near about the middle of the sequence and contributes to the red shift. A bigger shift in λ_max_ for an unfolded protein indicates a complete exposure on unfolding for a buried tryptophan. On the other side, a smaller shift signifies the tryptophan to be partially accessible for fluorescence in the folded state, which seems to be the case for the mutants. This was indicative of some structural rearrangement resulting in partial exposition of the interior tryptophan in the mutants vis-à-vis the wild type Sebox3.

### Structural changes in the interface from homology models

The modeled structure was aligned against other crystallized GGDEF proteins structures (4h54, 3tvk, 3qyy, 3ign) using PyMOL (The PyMOL Molecular Graphics System 2002). All these proteins aligned almost perfectly at their GGD(/E)EF regions. The GGD(/E)EF domain in VC0395_0300 is made up of a five-stranded beta sheet surrounded by multiple alpha helices and the GGEEF sequence occurs in the turn between the first two antiparallel strands of the sheet. This is in agreement with the three other structures which have been cited before (Lim et al. 2007; Tamayo et al. 2007; Gao and Stock 2009).

**Fig. 6:**
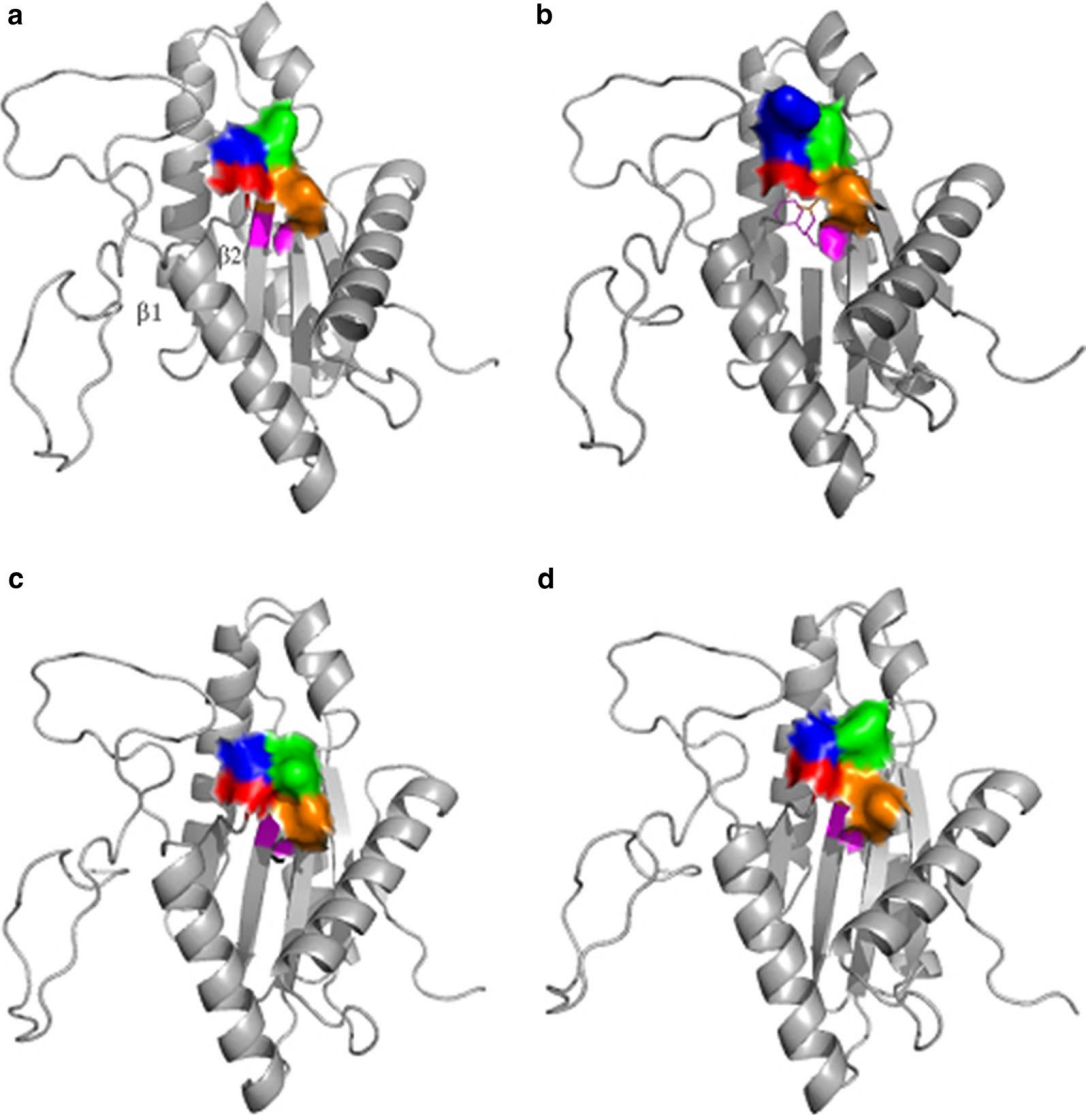
Differences in surface morphology in the GGEEF region of wild type (**a**), when the second G is mutated to R (Sebox5) (**b**), first E mutated to K (Sebox6) (**c**), second E mutated to K (Sebox7) (**d**). The first G is depicted in *red*, second G in *blue*, first E in *green*, second E in *orange*, and the F in *magenta*. The first and the second antiparallel strands of the beta sheet have been demarcated in **a**

Similarly, models were constructed for the mutant proteins Sebox5–7, and the model with the least free energy was chosen. All the generated models had more than 97 % of the residues in the favourable regions of the Ramachandran plot and the rest in the allowed regions. The mutant structures also show more or less similar architecture with regard to the domain, but, there is significant difference in the GGEEF turn between β1 and β2. This has been highlighted in Fig. 6.

While the structure of the PleD from *P. aeruginosa* and the XCC4471^GGDEF^ protein from *Xanthomonas campestris* showed the presence of compact interaction between the two consecutive, highly conserved glycines in the first two sites, generation of a G to R mutation in Sebox5 has disrupted the compactness which is essential for interaction with the c-di-GMP. Comparison of the surface morphology of the wild type and mutant proteins reveal significant differences in the surface of the wild type and the mutant Sebox5. The introduction of the lysine in place of the first glutamate in Sebox6 also changes the surface of the turn. It is to be noted that both Sebox5 and Sebox6 show a significant loss of biofilm formation abilities compared to the wild type. However, the lack of diguanylate cyclase activity in any of the proteins does not allow us a clear hypothesis.

## Discussions

*V. cholerae* possesses multiple sequences in the genome encoding GGD(/E)EF domain proteins, EAL proteins or both. One of them with a putative GGEEF domain, VC0395_0300 was chosen for characterization. To understand the effects of mutation at the GGEEF signature sequence, site-directed mutagenesis using the mega primer method was utilized to generate three mutants Sebox5, Sebox6 and Sebox7 representing mutations at the second G, first E and the second E, respectively. Both the wild type (Sebox3) and mutants showed considerable loss of cell viability, a trait often associated with recombinant GGD(/E)EF proteins. Functional characterization of the wild type protein showed significant biofilm formation, while the mutants had a remarkable loss of biofilm-forming ability, the most pronounced being Sebox6. Enzymatic assays to determine diguanylate cyclase activity did not reveal any evidence for the formation of c-di-GMP when the wild type and mutant proteins were allowed to react with the substrate GTP. Interestingly though, in vitro phosphodiesterase activity was demonstrated by Sebox3, 5, 6 and 7 despite the lack of an EAL or HD-GYP domain in the sequence. However, this confirmed the fact that the phosphodiesterase activity does not have any correlation with the GGEEF domain, as mutagenesis did not affect the phosphodiesterase activity. This is in line with newer findings of the activities and roles of GGD(/E)EF proteins from some unorthodox systems. Recent inputs by authors (Petrova et al. 2015) reiterate the diversity and complexity of diguanylate cyclases bearing GGD(/E)EF signatures in the regulation of biofilm formation apart from the conventional role of conversion of GTP to c-di-GMP.

Looking for an explanation to the loss of biofilm forming ability in the mutants as compared to the wild type, unfolding studies with guanidinium hydrochloride were undertaken and subsequent fluorescence were observed. The results point to some rearrangement in the architecture of the mutant proteins compared to the wild type as revealed by the shift in the emission maxima of tryptophan fluorescence. The homology derived model of the wild type protein showed structural familiarity with GGD(/E)EF domains from other structures. However, surface architecture of the mutants Sebox5 and Sebox6 show marked departure from the surface of the wild type GGEEF turn. From the observed properties, VC0395_0300 seems to be unique in its function, as it displays a GGEEF domain without a diguanylate cyclase activity, but shows a phosphodiesterase activity inspite of the absence of the EAL domain in the correct orientation. While we need to ascertain whether there is a GTP binding site in the protein or whether c-di-GMP binds to it at all, it is certainly a novel report of a GGEEF protein with phosphodiesterase activity. The shift in structure maybe postulated as a cause for the differences in biofilm forming abilities of the wild type versus the mutants, but other biophysical characterization would be necessary to fully understand the basis.
